# Risk for Prison-to-Community Tuberculosis Transmission, Thailand, 2017–2020

**DOI:** 10.3201/eid2903.221023

**Published:** 2023-03

**Authors:** Reiko Miyahara, Pundharika Piboonsiri, Boonchai Chiyasirinroje, Worarat Imsanguan, Supalert Nedsuwan, Hideki Yanai, Katsushi Tokunaga, Prasit Palittapongarnpim, Megan Murray, Surakameth Mahasirimongkol

**Affiliations:** National Institute of Infectious Diseases, Tokyo, Japan (R. Miyahara);; National Center for Global Health and Medicine, Tokyo (R. Miyahara, K. Tokunaga);; Harvard T.H. Chan School of Public Health, Boston, Massachusetts, USA (R. Miyahara, M. Murray);; The University of Tokyo, Tokyo (P. Piboonsiri);; Ministry of Public Health, Nonthaburi, Thailand (P. Piboonsiri, S. Mahasirimongkol);; TB/HIV Research Foundation, Chiang Rai, Thailand (B. Chiyasirinroje);; Chiangrai PrachChanukroh Hospital, Chiang Rai (W. Imsanguan, S. Nedsuwan);; Fukujuji Hospital and Research Institute of Tuberculosis, Kiyose, Japan (H. Yanai);; National Center Biobank Network, Tokyo (K. Tokunaga):; Mahidol University, Bangkok, Thailand (P. Palittapongarnpim);; Harvard Medical School, Boston (M. Murray)

**Keywords:** tuberculosis, TB, whole-genome sequencing, incarceration, prison, transmission, Mycobacterium tuberculosis, tuberculosis and other mycobacteria, Thailand, bacteria

## Abstract

To determine contributions of previously incarcerated persons to tuberculosis (TB) transmission in the community, we performed a healthcare facility–based cohort study of TB patients in Thailand during 2017–2020. We used whole-genome sequencing of *Mycobacterium tuberculosis* isolates from patients to identify genotypic clusters and assess the association between previous incarceration and TB transmission in the community. We identified 4 large genotype clusters (>10 TB patients/cluster); 28% (14/50) of the patients in those clusters were formerly incarcerated. Formerly incarcerated TB patients were more likely than nonincarcerated patients to be included in large clusters. TB patients within the large genotype clusters were geographically dispersed throughout Chiang Rai Province. Community TB transmission in the community was associated with the presence of formerly incarcerated individuals in Thailand. To reduce the risk for prison-to-community transmission, we recommend TB screening at the time of entry and exit from prisons and follow-up screening in the community.

Tuberculosis (TB) is a major public health problem in prisons globally. One meta-analysis reported that the incidence of TB among incarcerated persons was 4.1- to 26.9-fold higher than that in the general population (*1*). High levels of TB transmission in prisons have been attributed to crowding (*2*), poorly ventilated facilities (*3*), and lack of access to healthcare (*4*). In addition, several studies have reported a risk for spillover of TB from prisons into communities (*5*) and found that prisons can serve as drivers of population-level incidence (*6*–*8*). Evaluating the risk for TB transmission from prisons to the community is helpful for developing an effective intervention strategy to reduce the risk for spillover to the community.

In Thailand, the TB burden is high (*9*), and the country has the largest inmate population in Southeast Asia (411 inmates/100,000 national population in 2021) (*10*). A previous cross-sectional study conducted in a prison in Bangkok, Thailand, found that 46.5% of the population had latent TB infection diagnosed by tuberculin skin test or interferon-γ release assay (*11*).

Our objective with this study was to identify genotype clusters in the community by using whole-genome sequencing (WGS) data and to assess the contribution of previously incarcerated persons to these transmission clusters. The project was approved by the ethics committees of Chiang Rai Prachanukroh Hospital, Chiang Rai, and the Thai Ministry of Public Health. All TB patients enrolled in the study provided written informed consent.

## Methods

### Study Population

To evaluate host and pathogen genetic risk factors for TB development and transmission, during December 2017–February 2020, we conducted a healthcare facility–based cohort study in 18 districts in Chiang Rai Province, Thailand. We enrolled persons who had a positive *Mycobacterium tuberculosi*s culture, were >18 years of age, agreed to participate, provided blood or saliva samples for human DNA extraction, and were HIV negative at the time of TB diagnosis. Trained research nurses collected baseline demographic and clinical information, including age, sex, ethnicity, date of diagnosis, sputum test results, chest radiograph results, treatment outcome, education level, annual income, and incarceration history (including the year of entry into prison and the duration of incarceration). We did not enroll TB patients who were incarcerated at the time of TB diagnosis.

### WGS

**Figure 1:**
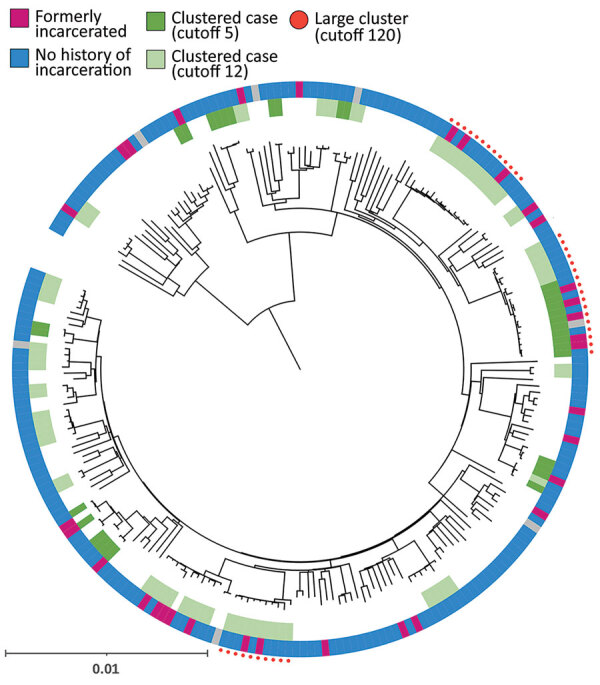
Phylogenetic tree of patients with pulmonary tuberculosis of *Mycobacterium tuberculosis* lineage in study of risk for prison-to-community tuberculosis transmission, Chiang Rai Province, Thailand, 2017–2020. Scale bar indicates 0.01 substitutions per site SNP, single-nucleotide polymorphism.

We extracted DNA from *M. tuberculosis* culture isolates and then sequenced the whole genomes by using Nextera XT (Illumina, https://www.illumina.com). We applied variant calling methods by using the H37Rv reference genome (GenBank accession no. NC_00962.3) (*12*). We used an in-house Python script to determine the *M. tuberculosis* lineage on the basis of WGS data (*13*). We constructed a phylogenetic tree by using the maximum-likelihood methods in MEGAX (*14*) and visualized the tree with the Interactive Tree of Life (iTOL) online tool, version 6.5.2 (https://itol.embl.de). We also analyzed pairwise single-nucleotide polymorphism (SNP) distances by using MEGA X and the frequency of pairwise SNP distances within sublineages (*12*). We used 2 SNP difference thresholds that have been used internationally to define clusters (*15*–*17*); the main analysis used a 12-SNP cutoff, which enabled inclusion of potentially related isolates, and the secondary analysis used a 5-SNP cutoff to identify highly related isolates. We defined large clusters as those that included >10 isolates linked to >1 other isolate by 12 pairwise SNP distances (Figure 1).

### Statistical Analyses

For persons for whom we had WGS and incarceration history data, we compared baseline characteristics based on the incarceration status by using χ^2^ tests for categorical variables. We used univariable and multivariable logistic regression models to evaluate the association between incarceration status and the risk for inclusion in a large cluster after adjusting for age, sex, ethnicity, and history of TB treatment. Because the only large clusters were clusters of lineage 2 strains, in the multivariate model adjusting for lineage we included only patients with lineage 2 isolates. We geocoded the patients’ addresses at the time of their TB diagnosis and used ArcGIS 10.0 software (Environmental Systems Research Institute, https://www.esri.com) to plot the addresses of patients in the same large clusters on a map. We used the nearest neighbor index (NNI), defined as the ratio of the observed mean distance to the expected mean distance, to assess whether the spatial distribution pattern was random (NNI = 1), dispersed (NNI >1), or clustered (NNI <1) (*18*). We performed statistical analyses by using Stata 16.0 (StataCorp LLC, https://www.stata.com).

## Results

A total of 10.1% (n = 60) healthcare facility patients had a history of former incarceration; those persons were more likely than other TB patients to be infected with lineage 2 *M. tuberculosis* strains, unemployed, 40–59 years of age, male, of hill tribe ethnicity, and to have a history of previous TB treatment (Table 1).

**Table 1:** Characteristics of nonincarcerated TB patients and formerly incarcerated TB patients, Chiang Rai Province, Thailand, December 2017–February 2020*

Characteristic	Nonincarcerated TB patients, n = 532	Formerly incarcerated TB patients, n = 60	p value
*Mycobacterium tuberculosis* lineage			0.006
1	256 (48.1)	15 (25.0)	
2	201 (37.8)	35 (58.3)	
3	6 (1.1)	1 (1.7)	
4	69 (13.0)	9 (15.0)	
Age, y			0.047
18–39	101 (19.0)	13 (21.7)	
40–49	99 (18.6)	18 (30.0)	
50-–9	139 (26.1)	17 (28.3)	
>60	60 (36.3)	12 (20.0)	
Sex			0.001
M	373 (70.1)	54 (90.0)	
F	159 (30.0)	6 (10.0)	
Ethnicity			0.032
Thai	408 (76.7)	37 (61.7)	
Hill tribe	115 (21.6)	15 (36.7)	
Other	9 (1.7)	9 (1.7)	
TB history			0.002
No	508 (95.5)	53 (88.3)	
Yes	24 (4.5)	6 (10.0)	
Unknown	0	1 (1.7)	
Prison year			
Before 1999	NA	11 (18.3)	
2000–2004	NA	5 (8.3)	
2005–2009	NA	15 (25.0)	
2010–2014	NA	16 (26.7)	
After 2015	NA	12 (20.0)	
Unknown	NA	1 (2.9)	
Prison time, y			
<1	NA	35 (58.3)	
1–2	NA	5 (8.3)	
>2	NA	20 (33.3)	

*NA, not applicable; TB, tuberculosis.

TB isolates were classified into 4 lineages: lineage 1 (271 [45.8%]), lineage 2 (236 [39.9%]), lineage 3 (7 [1.2%]), and lineage 4 (78 [13.2%]) (Figure 1). When we used the 12-SNP cutoff, the percentage of clustered cases was 6.6% (18 patients) in lineage 1, 46.2% (109 patients) in lineage 2, and 29.5% (23 patients) in lineage 4 (Appendix Table 1). None of the lineage 3 isolates were clustered. We identified 4 large clusters (>10 isolates) of strains with 150 (25.3%) isolates by using the 12-SNP cutoff and 1 large cluster with 33 (5.6%) isolates by using the 5-SNP cutoff (Table 2, Figure 1). The percentage of formerly incarcerated persons within the 4 large clusters was 28.0% (n = 14). In the univariate analysis for all lineages, *M. tuberculosis* isolates from previously incarcerated TB patients were 4.19 (95% CI 2.11–8.34-fold) more likely to be members of large clusters. After we adjusted for patient age, ethnicity, sex, and previous TB treatment history, prior incarceration remained associated with inclusion in large clusters (adjusted odds ratio [aOR] 4.47, 95% CI 2.05–9.32) (Table 2). Because the 4 large clusters included only lineage 2 isolates, we restricted our analysis to lineage 2 in the multivariate analysis. The odds ratio for prior incarceration decreased modestly (aOR 3.57, 95% CI 1.56–8.15) among lineage 2 isolates after adjustment for age, ethnicity, sex, and previous TB treatment history. Although there was only 1 large cluster with a cutoff of 5 SNPs, the genomic association with incarceration history was stronger than for persons in the large clusters with a cutoff of 12 SNPs (Appendix Table 2).

**Table 2:** Association between formerly incarcerated TB patients and *Mycobacterium tuberculosis* genotype clusters, all lineages, Japan, 2022*

Variable	Total	Unclustered	Small cluster, 2–6 cases	Large cluster, >10 cases	Unadjusted OR† (95% CI)	Adjusted OR‡ (95% CI)
Cutoff of 5 SNPs						
Total	592	559	22	11		
No history of incarceration	532 (89.9)	508 (91.0)	18 (81.8)	6 (54.6)	Referent	Referent
Formerly incarcerated	60 (10.1)	51 (9.1)	4 (18.2)	5 (45.5)	7.97 (2.36–26.96)	10.32 (2.47–43.09)
Cutoff of 12 SNPs						
Total	592	442	100	50		
No history of incarceration	532 (89.9)	407 (92.1)	89 (89.0)	36 (72.0)	Referent	Referent
Formerly incarcerated	60 (10.1)	35 (7.9)	11 (11.0)	14 (28.0)	4.19 (2.11–8.34)	4.47 (2.05–9.32)

*Values are no. (%) cases except as indicated. OR, odds ratio; SNP, single-nucleotide polymorphism; TB, tuberculosis. †ORs for the history of incarceration comparing those in large clusters with those unclustered or in small clusters. ‡ORs adjusted for age, ethnicity, sex, and history of TB treatment.

**Figure 2:**
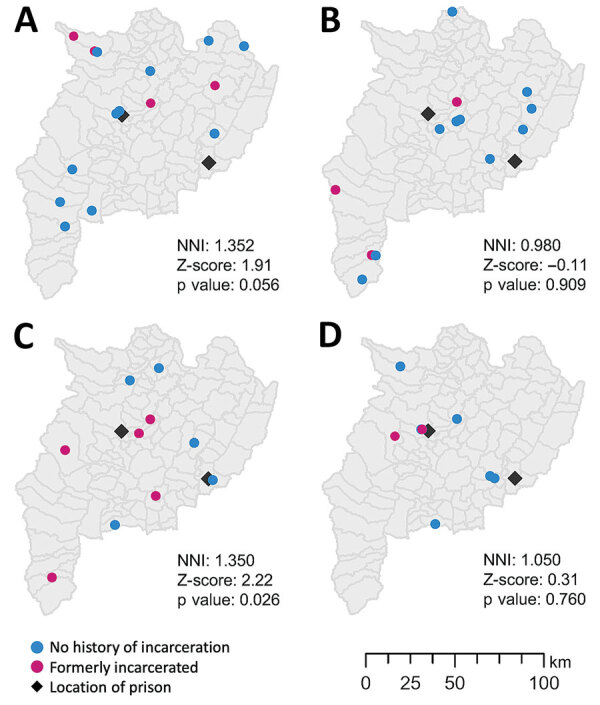
Distribution of clusters of tuberculosis patients in study of risk for prison-to-community tuberculosis transmission, Thailand, 2017–2020. NNI, nearest-neighbor index.

The proportion of formerly incarcerated persons in a large cluster with the 12-SNP cutoff ranged from 20.0% through 40.0% (Appendix Table 3). A maximum of 4/14 (28.6%) persons in the large clusters had received a TB diagnosis within 2 years after release. Cluster 3 included the highest percentage of formerly incarcerated persons (40.0%); 3 of the 4 formerly incarcerated patients had been incarcerated during 2014–2018, and TB developed within 2 years after they were released. We found little overlap between the periods of incarceration in clusters 1, 2 and 4. The NNIs were close to 1 or >1 (cluster 1, 1.352; cluster 2, 0.980; cluster 3, 1.350; cluster 4, 1.050), which suggested that the patients in the large clusters were not spatially clustered (Figure 2).

## Discussion

Our study found that formerly incarcerated TB patients were 4.7 times more likely than nonincarcerated TB patients to be linked with other patients in large transmission clusters. The association between being in a large cluster and having a previous incarceration history suggests that these genotypes could have circulated in prisons and spread to the community.

The timing of TB diagnosis varied after persons were released from prison. Only 29% of the formerly incarcerated persons in the large clusters received a TB diagnosis within the first 2 years after their release from prison.

Our results suggest that formerly incarcerated persons could be at higher risk for disease progression from latent to active TB for several years after their release from prison. A study from Brazil showed that among 83% of incarcerated persons in whom TB developed, diagnoses were within 2 years of release (*5*), and another study from Brazil estimated that it took 7 years for TB incidence rates among formerly incarcerated persons to decline to community levels (*6*). Because of the long-term risk for active TB development in incarcerated persons after they are released, careful follow-up of such incarcerated persons should be a focus of local public health centers in the community (*19*).

Our study showed that in this setting, formerly incarcerated TB patients were widely distributed throughout a large geographic area (Figure 2). This result was not consistent with that of a previous study in Lima, Peru, which found higher risk for multidrug-resistant TB among persons living near prisons (*20*). The difference in the spatial distribution of TB observed between the 2 studies is probably associated with the smaller number of more centralized prisons in Chiang Rai than in Lima. Lima has >10 million residents and 7 prisons, and formerly incarcerated persons are likely to reside in areas relatively close to the prisons in which they were incarcerated. In contrast, Chiang Rai Province has only 1.2 million inhabitants dispersed over ≈4,500 square miles and only 2 prisons. In addition, 1 study from Brazil found that prison-related TB genotypes were widely dispersed throughout regions because some inmates were transferred from one prison to another (*21*). With dissemination of the risk for TB infection throughout large areas, it would be challenging to detect links between formerly incarcerated TB patients and community TB patients and to estimate the effect of incarceration history on the community transmission.

TB control strategies in prisons should focus on not only reducing TB transmission in prison but also on preventing spillover from prison to community. In 2021, the World Health Organization Consolidated Guidelines on Tuberculosis updated the recommendation of systematic screening in prisons from a “conditional recommendation” to a “strong recommendation” but noted that there was “very low certainty of evidence” (*22*). As a recent modeling analysis showed (*6*), combined interventions could reduce TB incidence in prisons and in the general population. Options for preventing TB transmission in prisons include screening at the time of entry into the prison, periodic mass screening of incarcerated persons, or both. To control prison-to-community transmission, exit screening before persons return to their respective residential areas and follow-up testing in the community should also be performed (*6*). Furthermore, although the World Health Organization guidelines conditionally recommend preventive therapy in incarcerated persons depending on resource availability and the local risk for TB (*19*), few countries with low TB burden and high income have implemented preventive therapy programs for incarcerated persons (*23*). To reduce illness and death as well as the risk for TB transmission, the benefits of preventive therapy for incarcerated persons in countries with high TB burden should also be considered (*6*).

Among the limitations of our study, we were not able to establish exact and direct epidemiologic links between incarcerated persons and persons in the general population through contact tracing and are therefore not able to rule out the possibility that formerly incarcerated persons were infected in the community after release rather than vice versa. In addition, our study did not include all TB patients for whom diagnosis was made at the study site because we excluded patients who did not have culture isolates, who died before we contacted them, and who were co-infected with HIV. Therefore, we missed some links that might have affected the number of clusters, the size of clusters, and the proportion of formerly incarcerated persons in large clusters.

In conclusion, our study determined that large clusters included a high percentage of formerly incarcerated TB patients with variable years of incarceration and residential areas. Because prison-related genotypes are circulating in the community, control strategies such as entry and exit screening at release and follow-up screening in the community should be considered to prevent TB-associated illness and death among incarcerated persons and community transmission.

Appendix Supplementary information for study of risk for prison-to-community tuberculosis transmission, Thailand.
