# Supplementary material for: Risk for Prison-to-Community Tuberculosis Transmission, Thailand, 2017–2020
Source: Emerg Infect Dis. 2023 Mar;29(3):477–83. doi: 10.3201/eid2903.221023 (PMC9973682; doi:10.3201/eid2903.221023)
Supplement: Appendix — Supplementary information for study of risk for prison-to-community tuberculosis transmission, Thailand. [file 22-1023-Techapp-s1.pdf]

Article DOI: https://doi.org/10.3201/eid2903.221023

Risk for Prison-to-Community Tuberculosis Transmission, Thailand

Appendix

Appendix Table 1. Characteristics of pulmonary TB patients in Chiang Rai province

Variable	Category	Total	Unclustered		Small cluster (2-9)		Large cluster (over10)	
SNP differences			Cutoff 5	Cutoff 12	Cutoff 5	Cutoff 12	Cutoff 5	Cutoff 12
Total		592	559	442	22	100	11	50
Lineage	1	271 (45.8)	271 (48.5)	253 (57.2)	0	18 (18.0)	0	0
	2	236 (39.9)	203 (36.3)	127 (28.7)	22 (100)	59 (59.0)	11 (100)	50 (100)
	3	7 (1.2)	7 (1.3)	7 (1.6)	0	0	0	0
	4	78 (13.2)	78 (14.0)	55 (12.4)	0	23 (23.0)	0	0
Age	18-39	114 (19.3)	97 (17.4)	74 (16.7)	10 (45.5)	27 (27.0)	7 (63.6)	13 (26.0)
	40-49	117 (19.8)	113 (20.2)	82 (18.6)	2 (9.1)	22 (22.0)	2 (18.2)	13 (26.0)
	50-59	156 (26.4)	147 (26.3)	123 (27.8)	8 (36.4)	25 (25.0)	1 (9.1)	8 (16.0)
	≥60	205 (34.6)	202 (36.1)	163 (36.9)	2 (9.1)	26 (26.0)	1 (9.1)	16 (32.0)
Ethnicity	Thai	445 (75.2)	425 (76.0)	352 (79.6)	14 (63.6)	62 (62.0)	6 (54.6)	31 (62.0)
	Hill-tribe	137 (23.1)	125 (22.4)	85 (19.2)	7 (31.8)	34 (34.0)	5 (45.5)	18 (36.0)
	Others	10 (1.7)	9 (1.6)	5 (1.1)	1 (4.6)	4 (4.0)	0	1 (2.0)
Sex	Male	427 (72.1)	409 (73.2)	326 (73.8)	12 (54.6)	67 (67.0)	6 (54.6)	34 (68.0)
	Female	165 (27.9)	150 (26.8)	116 (26.2)	10 (45.5)	33 (33.0)	5 (45.5)	16 (32.0)
TB history	No	561 (94.8)	533 (95.4)	422 (95.5)	18 (81.8)	92 (92.0)	10 (90.9)	47 (94.0)
	Yes	30 (5.1)	25 (4.5)	19 (4.3)	4 (18.2)	8 (8.0)	1 (9.1)	3 (6.0)
		1 (0.2)	1 (0.2)	1 (0.2)	0	0 (0)	0	0 (0)
Incarceration	No	532 (89.9)	508 (90.9)	407 (92.1)	18 (81.8)	89 (89.0)	6 (54.6)	36 (72.0)
	Yes	60 (10.1)	51 (9.1)	35 (7.9)	4 (18.2)	11 (11.0)	5 (45.5)	14 (28.0)
Prison year (n=60)	Before 1999	11 (18.3)	11 (4.8)	7 (20.0)	0	2 (18.2)	0	2 (14.3)
	2000-2004	5 (8.3)	3 (1.3)	2 (5.7)	0	0 (0)	2 (18.2)	3 (21.4)
	2005-2009	15 (25.0)	14 (6.1)	12 (34.3)	0	2 (18.2)	1 (9.1)	1 (7.1)
	2010-2014	16 (26.7)	12 (5.3)	6 (17.1)	2 (9.1)	3 (27.3)	2 (18.2)	7 (50.0)
	After 2015	12 (20.0)	10 (4.4)	8 (22.9)	2 (9.1)	3 (27.3)	0	1 (7.1)
	Unknown	1 (1.7)		0		1 (9.1)		0 (0)
Prison days (n=60)	<1 year	35 (58.3)	29 (5.2)	18 (51.4)	4 (18.2)	10 (90.9)	2 (18.2)	7 (50.0)
	1-2year	5 (8.3)	3 (0.5)	3 (8.6)	0	0 (0)	2 (18.2)	2 (14.3)
	≥2years	20 (33.3)	19 (3.4)	14 (40.0)	0	1 (9.1)	1 (9.1)	5 (35.7)

Appendix Table 2. Association between formerly incarcerated TB patients and clusters, restricted to lineage 2 isolates*

Variable	Total	Unclustered	Small cluster (2-6)	Large cluster (≥10)	Unadjusted OR (95%CI)†	Adjusted OR (95%CI)‡
Cutoff 5						
Total	236	203	22	11		
No history of incarceration	201(85.2)	177 (87.2)	18 (81.8)	6 (54.6)	Reference	Reference
Formerly incarcerated	35 (14.8)	26 (12.8)	4 (18.2)	5 (45.5)	5.42 (1.56-18.86)	7.34 (1.64-32.92)
Cutoff 12						
Total	236	127	59	50		
No history of incarceration	201 (85.2)	114 (89.8)	51 (86.4)	36 (72.0)	Reference	Reference
Formerly incarcerated	35 (14.8)	13 (10.2)	8 (13.6)	14 (28.0)	3.06(1.42-6.58)	3.57 (1.56-8.15)

*OR, odds ratio; TB, tuberculosis.
†ORs for the history of incarceration comparing those in large clusters with those unclustered or in small clusters.
‡ORs adjusted for age, ethnicity, sex, and history of TB treatment.

Appendix Table 3. Characteristics of formerly incarcerated TB patients in four large clusters using 12 SNP cutoff*

Cluster	#	ΔSNP≤5	Age/sex	-2000	2001	2002	2003	2004	2005	2006	2007	2008	2009	2010	2011	2012	2013	2014	2015	2016	2017	2018	2019	2020
**Cluster1†**	1	○	40/M																					
5/17, 29.4%	2	○	30/M																					
	3	○	30/F																					
	4	○	40/M																					
	5	○	30/M																					
**Cluster2**	6		60/M																					
3/13, 23.1%	7		40/M																					
	8		40/M																					
**Cluster3**	9		60/M																					
4/10, 40.0%	10		30/M																					
	11		60/M																					
	12		30/M																					
**Cluster4**	13		40/M																					
2/10, 20.0%	14		40/F																					

*Dark gray: incarcerated, light gray: possibly incarcerated, yellow: year of TB diagnosis, ΔSNP≤5: Isolates linked at least one other isolate by no more than 5 pairwise SNP distances.
†Number of incarcerated TB patients/total number of TB patients, percentage of incarcerated people.
